# A High with Unexpected Air: Subcutaneous Emphysema in a Patient with Cannabinoid Hyperemesis Syndrome

**DOI:** 10.51894/001c.165622

**Published:** 2026-07-31

**Authors:** Ashley Kowynia, Ashley Yangouyian, Ashley Zuzelski

**Affiliations:** 1 Henry Ford - Jackson

## 89

### Introduction

Cannabinoid hyperemesis syndrome (CHS) is an increasingly recognized condition characterized by cyclic nausea and vomiting in the setting of chronic cannabis use. Although typically self-limited, severe and repetitive emesis can lead to rare but serious complications, including barotrauma with pneumomediastinum and subcutaneous emphysema. Early recognition is critical in the emergency department to exclude life-threatening causes such as esophageal perforation.

### Case Description

A 30-year-old female with a history of chronic cannabis use presented with 24 hours of persistent nausea, non-bloody emesis, abdominal pain, and constipation. She awoke with acute right-sided facial swelling after a day of repeated vomiting. Vital signs stable, afebrile. Examination revealed right-sided facial swelling from the lower eyelid to the mandible with palpable crepitus and mild erythema. Laboratory studies showed leukocytosis (WBC 22.1 ×10³/µL), lactate 2.8 mmol/L, and anion gap 15. Urine drug screen was positive for cannabis. She received intravenous fluids and empiric antibiotics (ceftriaxone, later broadened to piperacillin-tazobactam and vancomycin). Chest radiograph demonstrated subcutaneous air without pneumothorax. CT of the face, neck, and chest confirmed subcutaneous emphysema with pneumomediastinum. Thoracic surgery was consulted. Upper gastrointestinal contrast study was negative for esophageal perforation. The patient was managed conservatively with bowel rest, antiemetics, and close monitoring. Gastroenterology was consulted, and no intervention was required. Repeat chest radiograph the following day showed improvement in subcutaneous emphysema, and she was advanced to oral intake.

### Discussion

This case illustrates pneumomediastinum and subcutaneous emphysema as uncommon but clinically significant complications of forceful emesis in suspected CHS. The pathophysiology is consistent with barotrauma from markedly increased intrathoracic pressure leading to alveolar rupture and air dissection along fascial planes. Although spontaneous pneumomediastinum is often self-limited, distinguishing it from Boerhaave syndrome is critical, as esophageal perforation carries high morbidity and mortality. Leukocytosis and elevated lactate may confound the clinical picture and prompt broad-spectrum antibiotics and specialty consultation. Once perforation is excluded, management is typically conservative with bowel rest, supportive care, and counseling on cannabis cessation to prevent recurrence. This case emphasizes the importance of thorough evaluation, multidisciplinary collaboration, and awareness of rare complications of CHS in emergency practice.

